# Is sex ratio at birth an appropriate measure of prenatal sex selection? Findings of a theoretical model and its application to India

**DOI:** 10.1136/bmjgh-2017-000675

**Published:** 2018-07-19

**Authors:** Sylvie Dubuc, Devinderjit Singh Sivia

**Affiliations:** 1 Department of Geography, University of Reading, Reading, UK; 2 Saint John’s College, University of Oxford, Oxford, UK

**Keywords:** mathematical modelling, maternal health, medical demography, public health, health policy

## Abstract

Son preference and prenatal sex selection against females have resulted in significant sex ratio at birth (SRB) imbalances well documented in several Asian countries, including India and China. The SRB bias is generally used as indicator for the extent and trends of prenatal sex selection against females. Decreasing fertility levels are expected to increase sex selection and thus SRB bias, since desiring fewer children increases the risk for families to remain sonless (fertility squeeze effect). We developed and employ mathematical models linking family size, birth order and childbearing strategies with population SRB bias. We show that SRB bias can increase despite fewer sex selection interventions occurring, inconsistent with the expectation of the fertility squeeze effect. We show that a disproportionality effect of fertility reduction amplifies SRB bias, in addition to the fertility squeeze effect, making SRB bias an inaccurate indicator for changes in sex selection practices within a population. We propose to use sex selection propensity (proportion of couples intervening) to measure behavioural change and evaluate policies targeting sex selection practices. We apply our findings to India, showing for instance that sex selection propensity in Punjab and Delhi was lower than in Rajasthan or Uttar Pradesh, despite significantly higher SRB bias in the former. While we observe a continuous overall increase in the SRB over the 2005–2010 period in India, our results indicate that prenatal sex selection propensity started declining during that period.

**Key questions**

**What is already known?**

- Son preference and prenatal sex diagnostics have led to prenatal sex selection practices against females in Asian countries.
- The imbalance in sex ratio at birth (SRB) is generally used as indicator for the extent of prenatal sex selection.
- Decreasing fertility increases the likelihood of remaining sonless; therefore, more couples feel pressured to secure a male offspring through prenatal sex selection.
- The quantitative relationship between fertility and SRB remains unclear.

**What are the new findings?**

- Using mathematical models we quantitatively relate fertility and SRB with the propensity of prenatal sex selection, thereby identifying a ‘disproportionality effect of fertility on SRB bias’.
- SRB bias is not a reliable indicator for the extent of prenatal sex selection practices and related policy making.

**What do the new findings imply?**

- We propose to use sex selection propensity as reliable indicator for prenatal sex selection practices, allowing to measure and meaningfully compare the extent of prenatal sex selection across regions and over time, and to evaluate policies.

## Introduction

Prenatal sex selection against females (PSS) is generally evidenced by a masculinisation of the sex ratio at birth (SRB; the ratio of boys to girls). It has been reported in South Korea,^1^ China,^2^ India,^3 4^ and more recently in Vietnam,^5^ Nepal^6^ and the Caucasus.^7^ SRB bias has also been found in Western countries with substantial Asian diasporas, notably in the UK,^8^ the USA and Canada.^9–11^ Substantially contributing to the masculinisation of the juvenile sex ratios since the 1980s, PSS is thought to have led to more than 30 million ‘missing’ female births, mostly in Asia, with an estimated 1.7 million in 2015 alone.^12^ Hitherto, PSS is thought to have largely resulted from fetal sex determination (mainly ultrasound), followed by the abortion of female fetuses. More recently, PSS has also become technically possible through advances in medically assisted reproduction techniques^i^.^13 14^

Many policy interventions at the local, regional or national levels have been introduced^ii^ to reduce sex selection against females. The continuous rise in the SRB imbalance has been interpreted as evidence of prenatal sex selection diffusion and a failure of such policy interventions.^6 15–19^ However, other researchers argue that without legislation, the SRB bias would have likely increased.^20^ In India, the continued rise in sex imbalances seen in the 2001 census engendered more restrictive legislation on access to prenatal sex selection methods (Pre-Conception and Pre-Natal Diagnostic (PNDT) Act, 1994, revised in 2003) and introduced alternative policies, including pregnancy tracking and monitoring schemes, child protection schemes and the multiplication of conditional cash transfer (CCT) schemes.^18 21^ The efficiency of such policy interventions remains difficult to evaluate. For example, the interagency statement ‘Preventing gender-biased sex-selection’ (Office of the High Commissioner for Human Rights (OHCHR), United Nations Population Fund (UNFPA), Unicef, UN Women and WHO) called for the development and use of indicators for tracking change and the impact of interventions.^22^

In countries like India, where the fertility transition is well under way, the dual desire for small families and male offspring exerts strong pressure on sonless parents to select for a son.^1 4 23^ This is because the probability of remaining sonless, when left to chance, increases exponentially with fewer children.^8^ Guilmoto^24^ introduced the concept of the ‘fertility squeeze’ whereby, with fewer desired children, the cost of having additional children until male offspring is achieved by chance becomes decreasingly acceptable (expressed as acceptable proportion of female births, APFB), resulting in more parents reverting to sex selection and at lower birth order. Similarly, evidence in South and East Asia has shown that the distortion in the SRB is particularly pronounced at higher birth orders, when only daughters were born previously.^1 2 25–27^ A recent study by Jayachandran,^28^ based on survey data modelling, estimated that fertility reduction could explain up to half of India’s sex ratio increase over the 1981–2011 period. A number of studies have further evidenced the male-preferring stopping rule of childbearing,^29 30^ where parents of daughters only are more likely to progress to the next parity in an attempt to achieve a male birth and stop childbearing after a son is born, resulting in a strong male-biased sex ratio at last birth.^31 32^

Gender imbalances at birth provide the only readily available (indirect) method to evidence PSS quantitatively. However, the SRB is problematic for evaluating potential changes in sex selection attitudes and practices because desired family size influences reproductive behaviours associated with son preference,^4 31 33^ so that the extent of sex selection practices in a population cannot be directly evidenced from SRB bias.^24^

We were interested in estimating how many couples would have to sex-select in order to account for SRB bias sex selection in populations with varying fertility levels. We modelled the relationship between fertility and sex selection propensity (the proportion of intervening couples) to explain SRB bias, taking into account the fertility squeeze, gendered parity progression and the male-preferring stopping rule. Importantly, we demonstrate that SRB bias is hypersensitive to changes in fertility, because the fertility squeeze acts synergistically with a disproportionality effect of fertility on SRB. We illustrate this for India, based on reported fertility and sex ratio imbalances. We show that national trends and regional patterns of sex selection propensity differ markedly from SRB trends and patterns. Our findings call into question the interpretation and use of SRB to inform and evaluate policy and reproductive medical practices. Alternatively, we propose a measure of sex selection propensity as a more direct and relevant indicator of the population at risk to sex-select, indicating how widespread the practice is and to monitor behavioural change over time.

## Methods

### The theoretical model

We developed probabilistic models, making assumptions regarding the order of intervention depending on the gender composition of previous siblings to integrate the fertility squeeze and differential childbearing stopping rule that can impact SRB outcomes. Initially, we designed a model assuming universal son preference and unconstrained access to sex selection technologies in a given (theoretical) population. We assumed interventions would occur at a single birth order (online supplementary appendix) in order to facilitate analytical progress in understanding the demographic mechanisms linking micro and aggregated levels. We used a simple model to account for the fertility squeeze effect, formulated as follows: Let the average number of children per family be λ (table 1) and the natural propensity for female births be *p.* Since the fertility squeeze effect cannot be directly observed, we introduce a latent variable, *n*, which is directly proportional to the aforementioned APFB (APFB=100−(1/n*100); Guilmoto 2009).^5^ In the model, *n* expresses an intervention threshold, that is, at what birth order a couple would sex-select to ensure a male offspring. Families with *n* or more children, but no son, would seek medical intervention to ensure that at least one birth is male.

10.1136/bmjgh-2017-000675.supp3 Supplementary data

**Table 1:** Description of the model variables

Variables	Description
R	Proportion of male births; sex ratio at birth.
λ	Average number of children per family within a population.
Φ	Proportion of intervening couples, that is, couples reverting to sex selection.
Ψ	Proportion of intervening parents (excluding childless couples).
n	Birth order threshold of intervention: universal threshold in model A; minimum universal threshold in model B.

Next, we accounted for the facts that (1) parents are likely to intervene at different birth orders, likely depending on their desired number of children; and (2) parents using sex selection to ensure a son most frequently stop childbearing thereafter.^27 32^ Therefore, we introduce the stopping rule (online supplementary appendix), that is, all parents would seek intervention only at last birth if sonless^iii^. This constraint generates a varying distribution of intervention thresholds, accommodating any combination of fertility and SRB levels. Instead of the universal threshold above, *‘n’* becomes the minimum birth order at which parents would intervene; for instance, for n=2, families with a total of two children and where the first child was a girl would have sex-selected for the second and last child. This allowed analysis of the interrelation between sex selection, average family size and SRB distortion in a population, independent of factors limiting son preference or its implementation.

We assume *in vitro* fertilisation-like intervention throughout, so that all sonless parents at *n*-1 births would intervene at the next birth. In case of female-selective abortion, only women carrying a female fetus would opt for intervention and about half (only those with a diagnosed female fetus) would have to resort to more than one intervention, ultimately resulting in a similar number of interventions as per using a preconception method (see online supplementary appendix). We used the Poisson distribution to model the distribution of the number of children, *N*, in families when given only the mean value, λ; the case of λ=1.7 is illustrated in online supplementary figure S1A. We assigned the probability of having *G* girls given *N* and *p*, using a binomial distribution as only two possible outcomes at each birth, a boy or a girl, are possible; the case of n=4 and p=0.486 is illustrated in online supplementary figure S1B. The value of p=0.486 was chosen for consistency with the norm of around 946 girls for every 1000 boys at birth (or 105.7 boys per 100 girls),^34 35^ and follows from the requirement that *p*/(1−*p*)≈0.946.

10.1136/bmjgh-2017-000675.supp1 Supplementary data

Having specified the model, the expected SRB (*R* in equations and figures) can be calculated for different values of λ and *n* (with *p* fixed at 0.486) by using equations (9), (10) and (18) derived in online supplementary appendix. The corresponding formula for the proportion of couples needing to undertake a gender-selective intervention (ie, the intervening fraction denominated as Φ) to ensure that the *n*^th^ child is male if the first *n*−1 births were female(s) is given in equations (11) and (19). Φ denotes the maximal proportion of intervening couples as each couple is assumed to potentially intervene once to account for aggregate SRB bias. Consequently, Φ also allows ready calculation of the prevalence of sex-selective interventions (absolute number of interventions), irrespective of multiple interventions by couples.

### Application to India

We applied our model to India using reported SRBs and fertility levels, providing novel measures of sex selection propensity over time and across states, using equation 19 (online supplementary appendix). We used United Nations data on SRB and total fertility aggregated at the national level over 1970–2010 and calculated the corresponding proportion of couples intervening and analysed trends in sex selection propensity. Finally, we used reported values of SRB (*R*) and total fertility (λ) by key Indian states provided by the national Sample Registration System (SRS) to calculate the proportion of couples intervening and mapped the geographical distribution of sex selection prevalence.

## Results

### Theoretical results

**Figure 1:**
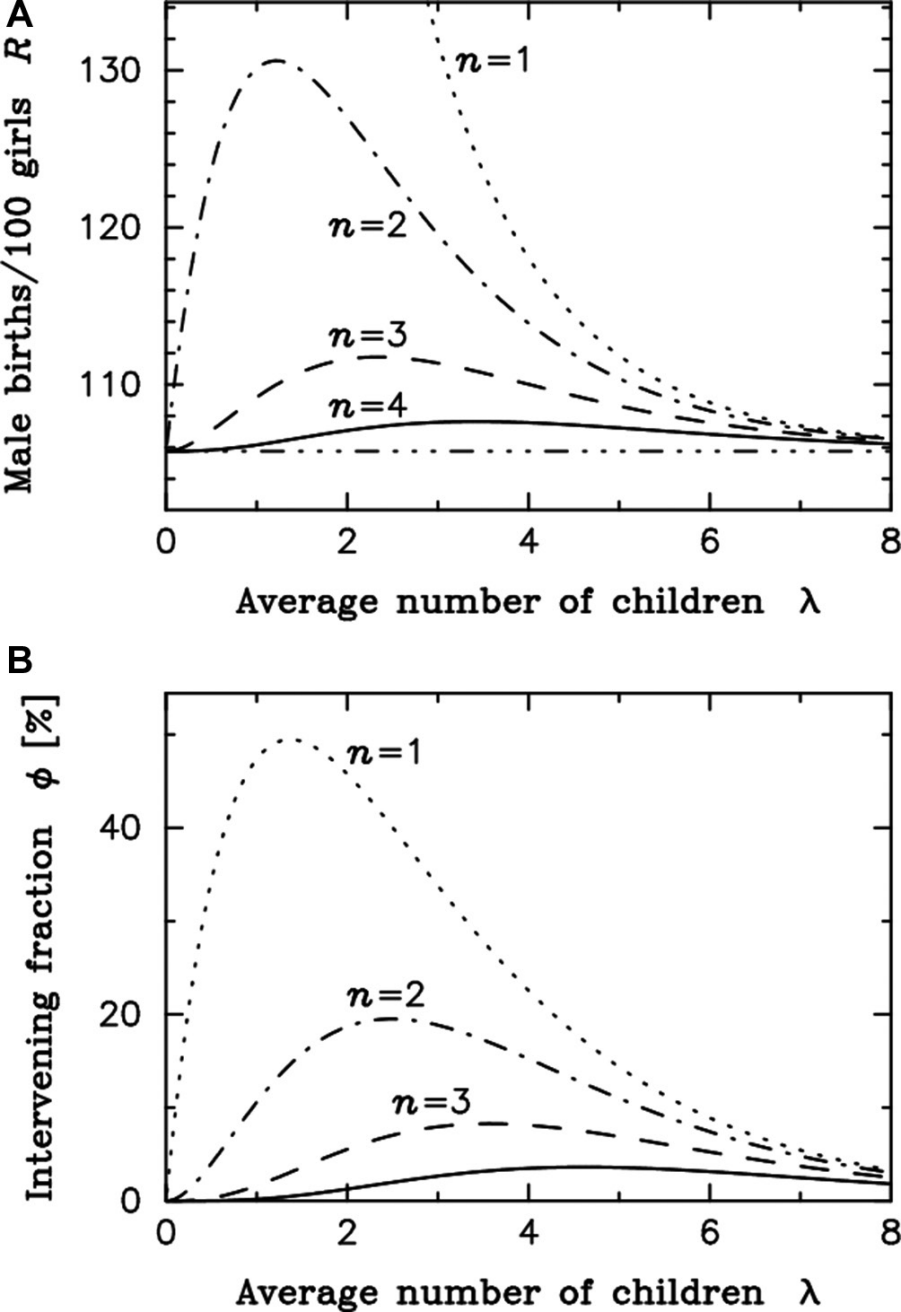
The stopping rule model. (A,B) n is the minimum threshold (birth order) at which parents would use sex selection if necessary to ensure a son. Moving down from the top left-hand corner, the four curves show the cases for n=1 (dotted), n=2 (dot-dash), n=3 (dashed) and n=4 (solid). For instance, at minimum intervention threshold n=2, the distortion in the sex ratio at birth (R) is increasing continuously with a reduction in the average number of children in the population (λ) until it reaches its peak value of 130.6 boys per 100 girls at λ equal to 1.196. Below that point, there is increasingly less couples reaching the intervention threshold of two children, explaining that the sex ratio bias is decreasing. The fraction of intervening couples initially increases with fertility reduction to a maximum of 19.5% when reaching about 2.5 children in average before starting to decrease with further fertility reduction.

The general nature of the dependence of *R* and Φ on the average size of the family (λ) for different sex selection thresholds is shown in figure 1. In accordance with the fertility squeeze hypothesis, the model shows that generally the SRB (*R*) and the proportion of couples intervening (Φ) increase with decreasing birth order threshold of sex selection couples opt for. *R* is little distorted when the average number of children is extremely low in figure 1A, and consistently figure 1B shows how the proportion of couples reverting to sex selection, Φ, goes to 0 for all threshold *n* when λ is very small. This is because most families are then childless and never reach the critical intervention threshold (*n*). Consequently, the distributions of Φ and *R* as a function of λ both exhibit a maximum (see figure 1 for n=3 and below). We observed a sharp increase in *R* with fertility reduction until *R* and Φ reach their maxima, illustrating that the SRB (*R*) is highly sensitive to changes in fertility rates within a population. The decreasing Φ with higher λ is expected because parents desiring many children are most likely to have a son without sex-selecting (figure 1B).

Surprisingly, figure 1 shows an initial rise in the distortion of the sex ratio as the average number of children declines, for any given *n*, even though the proportion of couples using gender-selective intervention diminishes. Indeed, the peak of sex ratio distortion occurs at a much lower average number of children per family than does the peak of interventions (figure 1), showing that with fewer children overall, a greater distortion in the SRB (*R*) may occur despite a reduction of the proportion of couples intervening (Φ) in a given population. This outcome is inconsistent with the fertility squeeze effect alone, which is expected to act proportionally on the fraction of couples intervening.

Our models include all couples, including those with no children. A decrease in the average number of children means more couples are childless, which could potentially significantly shift the Φ maxima position. Therefore, to exclude the possibility that the inclusion of childless couples accounts for the surprising result demonstrated in figure 1, we calculated the proportion of intervening parents (Ψ), instead of couples, and found a proximal maxima for Φ and Ψ, excluding this possibility, as shown for the n=2 scenario in online supplementary figure S2. The increase in childlessness with fertility reduction contributes to lower Φ to some extent, but excluding childless couples still results in a discrepancy between the maxima of the SRB and the maxima of the proportion of parents intervening (Ψ) on the fertility scale. Therefore, a yet unaccounted factor must impact on SRB bias.

10.1136/bmjgh-2017-000675.supp2 Supplementary data

#### The disproportionality effect of fertility on the SRB

**Figure 2:**
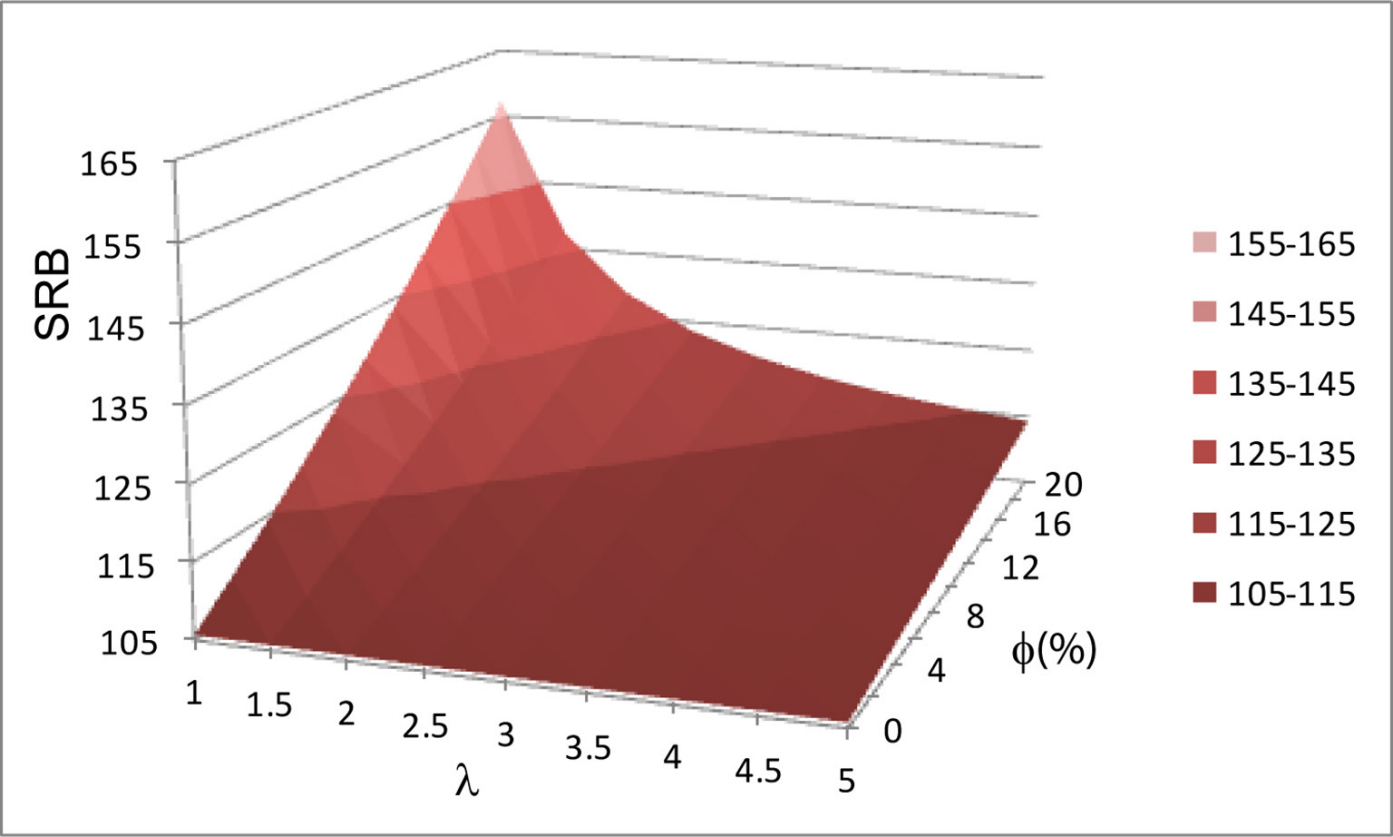
The disproportionality effect of fertility on the sex ratio at birth (SRB), R. R increases with an increasing proportion of intervening couples within a population (Φ, 0–20% shown). R also increases with a decreasing average number of children within the same population (λ). Note that for any constant Φ>0, R still increases with decreasing λ.

In order to disaggregate the impact of the fertility squeeze effect from other potential fertility influences on SRB, we computed the relationship between λ, Φ and *R* directly, using equation 19 (online supplementary appendix). Figure 2 shows that at any given constant Φ>0, the SRB (*R*) increases exponentially with decreasing fertility (λ), illustrated for 4%–20% of sex-selecting couples (Φ). We term this the *disproportionality effect* of fertility on the SRB, which is explained by the fact that within a small birth cohort, the number of ‘extra boys’ (or ‘missing girls’) weighs disproportionally on the resulting SRB. Importantly, the disproportionality effect impacts on the SRB independently of the fertility squeeze, so that both effects combined are cumulative (conceptualised in figure 5).

Practically, the disproportionality effect makes SRB an inadequate indicator to evaluate sex selection trends. In contrast, Φ is independent from the disproportionality effect and can be readily understood as the prenatal sex selection propensity in a population, reducing the complexity of inferring behavioural change in sex selection practices. Φ is directly proportional to the prenatal sex selection prevalence, that is, the absolute number of interventions, providing a measure of relative prevalence of sex selection, better suited to investigate changes in prenatal sex selection practices than SRB. We calculated Φ to re-evaluate SRB trends in India and illustrate our theoretical findings.

### Sex selection prevalence and change in India

**Figure 3:**
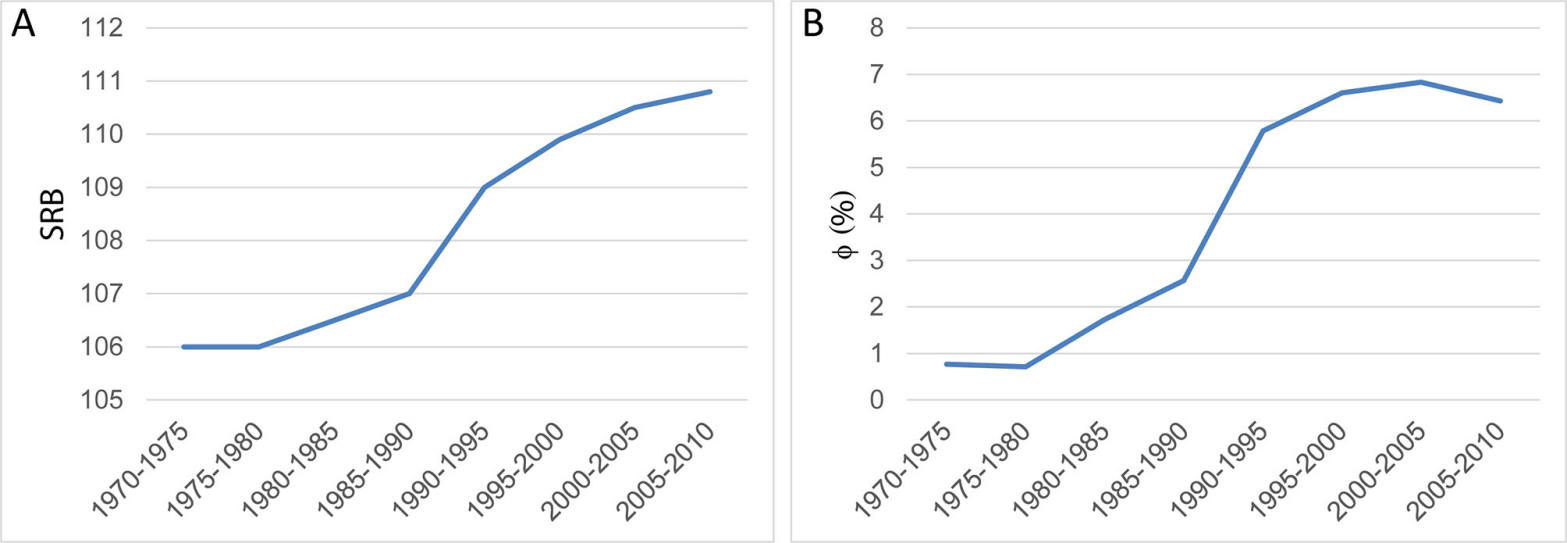
The sex ratio transition in India. (A) SRB national trends, 1970 to 2010. (B) National trends in prenatal sex-selection propensity, 1970 to 2010. Source: UN World Prospect database (observed R and λ). SRB, sex ratio at birth.

In India, fertility transition is well advanced, although with much regional variation. The continuous decline in fertility levels (average number of children per family) combine with an increasing bias in the SRB observed since the 1980s to the last census in 2011.^36^ Using SRB and total fertility between 1970 and 2010, we calculated the corresponding proportion of couples reverting to sex selection (Φ) (figure 3). We found that the propensity to sex-select (Φ) accelerated in the 1990s before reducing intensity, consistent with empirical findings by Jha and colleagues.^37^ Despite a continuous increase in the SRB until 2010 (figure 3A), sex selection propensity (Φ) reached its peak at the turn of the 21st century, before starting to decline (figure 3B). This finding challenges assumptions that sex selection has continued to increase in the last decade in India (see Discussion and conclusions section).

**Figure 4:**
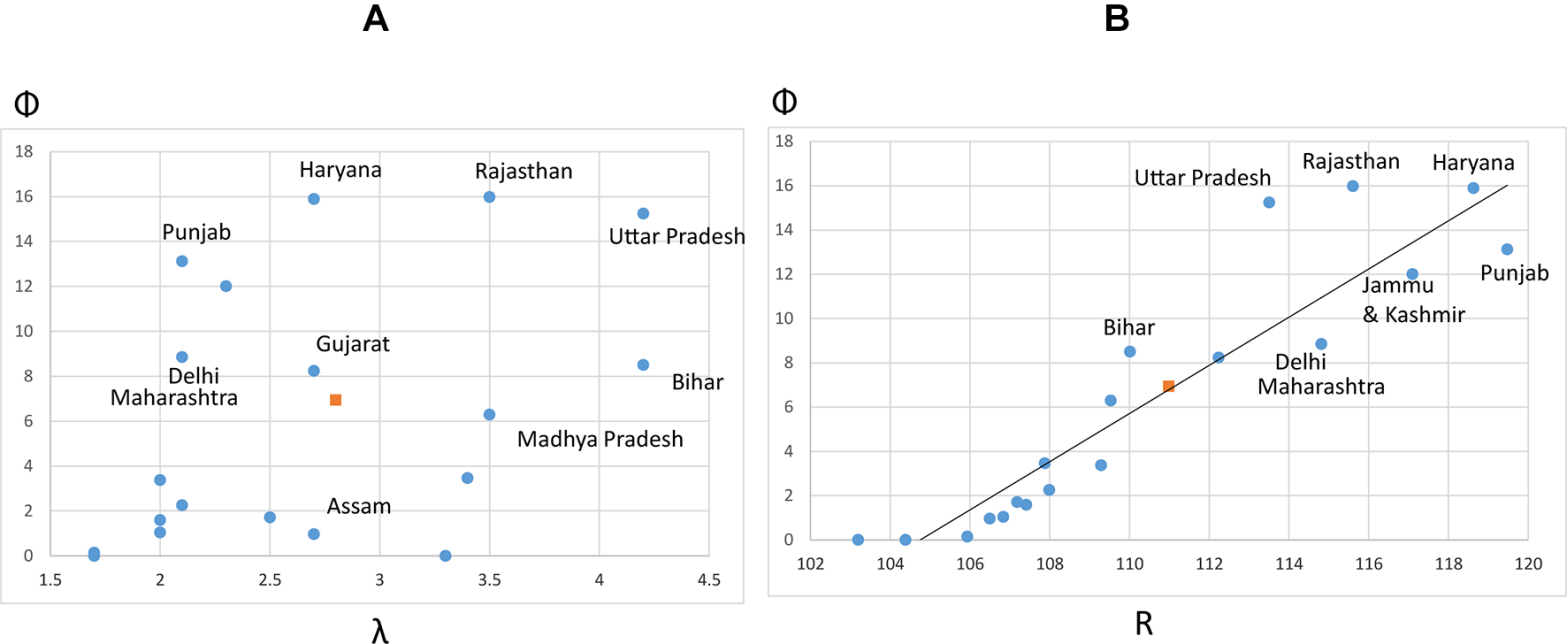
Sex selection prevalence across the main Indian states, in relation to reported fertility level (total fertility rate; TFR) and SRB, 2005–2007. (A) Relationship between reported fertility level (λ) and calculated proportion of couples using sex selection (Φ). λ is estimated using reported period total fertility rates. (B) Relationship between the reported SRB for the main states of India in 2005–2007 with the proportion of couples using sex selection (Φ). The fitting line represents the expected values of Φ, given R, based on the average relationship between Φ and R across the main Indian states (solid dots). States above the fitting line (eg, Rajasthan, Uttar Pradesh) have a higher sex selection propensity Φ than expected given their reported SRB. Those below the fitting line have in contrast a lower measured Φ than expected considering their SRB. Indian averages in squares. Source: Indian Sample Registration System Statistical Report 2011, tables 15 and 16 (http://www.censusindia.gov.in/vital_statistics/SRS_Reports.html). SRB, sex ratio at birth.

Beyond national trends, we observed pronounced regional differences in fertility and SRB. Rajan *et al* 36 measured an overall increasing bias in the national SRB between the last two censuses (2001, 2011). They also found that the SRB bias was stabilising or even reducing in some northern states, where historic trends of excess girls’ mortality and high levels of daughter deficit at birth are well known, but was worsening in other parts of the country. Using SRS 2005–2007 3 years’ average estimates, at the peak of SRB bias in northern India according to the SRS data^iv^, figure 4A shows how the ratio of intervening couples (Φ) relates to fertility across states. We found that sex selection propensity in the population was highest in Rajasthan, Haryana, Uttar Pradesh and Punjab, which exhibit different fertility levels (from 2.1 in Punjab to 4.2 in Uttar Pradesh).

Punjab’s highest SRB bias among Indian states is notorious, although in the most recent years it has reduced to levels below Haryana state according to the 2011 census data^v^. What is of particular interest is that even at its highest SRB bias (figure 4B), Punjab was not the state recording the highest sex selection propensity (figure 4A); the proportion of couples using sex selection was higher in Rajasthan and Utter Pradesh in 2005–2007 (about 16%), despite a less biased SRB compared with Punjab. The line fit in figure 4B shows the average relationship between SRB (*R*) and the intervening fraction (Φ). Those states positioned below the regression line have a lower Φ than the regression to the mean would expect, given their SRB. The disproportionality effect of fertility explains the departure from the mean in figure 4B; the distortion in the SRB is exacerbated in Punjab and Delhi, where fertility is low. In contrast, the effect of each single sex selection weighs less with larger average family sizes in Rajasthan and Uttar Pradesh.

At comparable reported levels of fertility, differences in the proportion of couples intervening can be due to differences in son preference across states and/or additional factors preventing sex selection. For instance, because Punjab and Delhi show similar fertility levels, their difference in Φ (figure 4A) suggests stronger son preference in Punjab than Delhi, assuming comparable levels of access to the technology in both states.

## Discussion

### The hypersensitivity model of fertility on SRB

**Figure 5:**
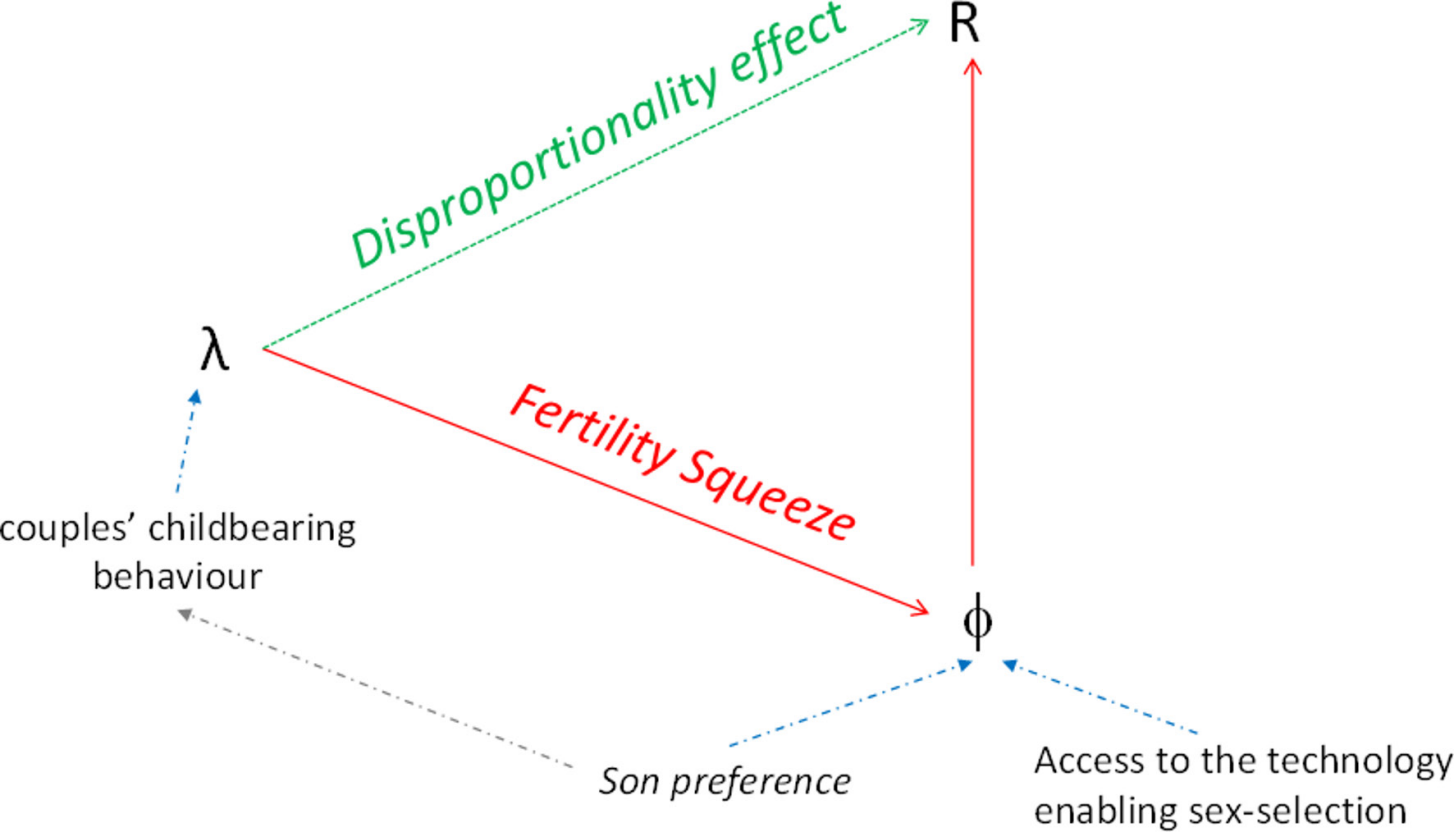
The macro impact of behavioural change: fertility squeeze and disproportionality effects on the sex ratio at birth.

Our model elucidated the demographic micro–macro dynamics between PSS and changes in SRB (figure 5). We show that, at low fertility levels, a small proportion of sex-selective procedures suffice to significantly distort the SRB, and importantly an increase in sex ratio bias does not necessarily equate to an increase in sex selection events. This is inconsistent with the fertility squeeze effect. Consequently, the SRB is not an appropriate indicator to evaluate behavioural changes. Figure 5 conceptualises our findings, linking childbearing behaviour and aggregated SRB in a population. Given constant son preference, if more couples want fewer children, more families are at risk of remaining sonless. The relationship between fertility (λ) and the proportion of intervening couples (Φ) reflects the fertility squeeze effect.^24^ The resulting higher proportion of intervening couples (Φ)—and intervening at lower birth order—increases the SRB bias, with the relationship between Φ and SRB being linear (figure 2).

Perhaps counterintuitively, we show that when the number of children per family is decreasing on average, the SRB bias may still increase *despite* a *decreasing* proportion of parents seeking sex-selective intervention. This is because, as we have shown, the impact of each sex selection event on distorting the aggregated SRB varies according to the level of fertility and increases with a reduction of average family size in an exponential relationship. This disproportionality effect is independent of the fertility squeeze effect.

In other words, even if the fertility squeeze effect would not increase the proportion of intervening couples, the SRB would still increase when fertility declines, due to the disproportionality effect. In practice, both effects apply synergistically to distort the SRB. Because both effects follow an exponential function in relation to average family size, the SRB is hypersensitive to levels and change in fertility (figure 2), with important implications for interpreting the SRB. For example in India, the increase in SRB, especially exacerbated in districts well engaged in the fertility transition,^15^ is due to the macro-level disproportionality (figure 4). In contrast to the SRB, the proportion of intervening couples (Φ) is not affected by the disproportionality effect and is readily interpretable to measure PSS trends. We used Φ, that is, the percentage of sex-selecting couples accounting for SRB bias, to re-evaluate trends of PSS in India. Φ is also directly proportional to the ‘missing girls’ as an expression of sex selection prevalence in a population.

### Diffusion of the technology versus disproportionality effect of fertility reduction

Previous empirical research measured a weakening of son preference despite SRB remaining high and even increasing in India^25^ and China.^38^ To explain this apparent paradox, Bhat and Zavier suggested that increased diffusion of sex selection technology could account for this, enabling more (prospective) parents to implement their preferences, despite an overall weakening of gender preference.^25 32^ This has led observers to recommend further restricting access to sex selection technology^38^ to control and limit families’ opportunities to implement their preferences. Our results offer an alternative explanation. We show that even without increase in sex selection prevalence and even with fewer sex-selective interventions, the SRB may continue to increase, due to the disproportionality effect of fertility reduction, providing quantitative support to suggestions of weakening son preference overall in India.^39^ One possible limitation of the new proposed indicator (Φ) is that it requires setting a ‘natural’ SRB benchmark. Several close empirical observations have been proposed in the literature, between 105 and below 106 male births per 100 female births, and some variation across world regions have been documented^vi^. This has no implications for our theoretical demonstration, but in applied research the calculated proportion of intervening couples would vary slightly depending on the benchmark value adopted. However, importantly, the adoption of a unique benchmark makes Φ a reliable indicator of sex selection propensity in a population and fully comparable across countries, subregions and over time.

A reversal in sex selection practices has been identified where a reduction in the SRB has been observed, as in South Korea from the late 1990s,^40 41^ or in some cities and regions of China and India.^36 42 43^ We demonstrate that in a context of fertility transition, a reversal of sex selection practice precedes the reduction in sex ratio bias. This modifies our understanding of the processes underlying gender population imbalances in Asia as related to development. For example, it challenges the widely held idea that the higher SRB observed among urban and educated women in many studies^6 37 44–46^ is due to more sex-selective interventions among these groups. It was proposed that despite weaker son preference among wealthier, educated urban women,^25 42 47^ they would have easier access to sex selection procedures to implement their preference compared with rural and lower socioeconomic groups. We show that this is not necessarily the case; educated urban women with smaller families on average would be characterised by the highest SRB bias even if they are not using sex selection more frequently than other socioeconomic groups. The gap between rural and urban population SRB bias has narrowed in the last decade in India, but some fertility differences remain. This suggests a higher prevalence of sex selection practice in rural areas, calling for further investigation.

### Measure of sex selection propensity to assess intervention

Our findings have implications for estimating sex selection as well as evaluating policy interventions targeting gender bias. Inferring behavioural change from the masculinisation of the SRB in a context of fertility transition is likely misleading because it is largely due to a scaling (disproportionality) effect. Despite a continuous increase in the SRB and the diffusion of sex selection technology^vii^,^17 25^ we measured a decrease in the recent trend in sex selection propensity in India (figure 4). This points to a significant weakening of son preference and suggests that policy intervention may have been effective in curbing sex selection and raising daughters’ valuation, although the impact of specific schemes remains to be evaluated. For example, in an evaluation of CCT schemes to improve girl children’s status in Haryana, Krishnan *et al* found, despite an improvement in girls’ immunisation, an increasing bias in the SRB over the study period (sample across 28 villages), producing an apparent paradox.^48^ However, increasing SRB bias does not necessarily indicate a failure of the intervention to limit sex selection. In fact, fewer couples might be using PSS if fertility has reduced. An estimation of the propensity of sex selection (Φ), derived from the combined SRB and fertility levels within the population surveyed, would be a more reliable indicator to evaluate the impact of interventions to promote the girl child.

Sex selection propensity and the proportional sex selection prevalence (ie, the total number of sex selection interventions) directly indicate the risk and spread, respectively, of sex selection interventions. Because policy interventions aiming to reduce sex selection and SRB bias act on Φ—either by reducing son preference (eg, child protection schemes) or access to sex selection technologies (eg, PNDT)—we recommend using trends in sex selection propensity (Φ) to inform the medical profession and assess policy interventions. Further, our findings suggest that policy initiatives targeting changes in gender representations and preferences to address sex ratios imbalances should be encouraged. Nonetheless, SRB remains an appropriate indicator for future population sex imbalances, important for concerns about future societal impacts of the marriage market squeeze.^49 50^

What would happen in a scenario of increasing fertility? Although the impact of the recent relaxation of the ‘one child policy’ on increasing fertility in China remains uncertain, if Chinese fertility rises in the near future, this will help reduce SRB imbalances. However, assuming continuous access to intervention methods without a change in gender preference, the SRB bias would reduce without reduction in sex selection prevalence or even despite an increase in the number of sex selections.

## Footnotes

i PSS is now possible through advances in medically assisted reproduction techniques, in particular in vitro fertilisation combined with prefertilisation selection of male spermatozoa (sperm sorting) or preimplantation genetic diagnosis and selection of male embryos.

ii See UNFPA 2014 and references therein.

iii This remains an idealised model; possibly not all parents would stop childbearing after intervention, for instance in the case of the death of a child/son or because some parents may want more than one son (although empirical evidence suggests that these circumstances are increasingly unlikely where fertility transition is advanced).

iv Analyses of the Sample Registration System data suggest that the peak in SRB bias is around 2005 in some northern states now showing a relative decrease.

v The accuracy of the very high sex ratio bias at a young age in Jammu and Kashmir reported in 2011 census data is much uncertain (eg, UNFPA report 2014).

vi Notably the ratio of boys to girls at birth is slightly lower overall in Africa (at about 103 per 100 girls).

vii Counteracting the diffusion of medical equipment, enforcement of the law potentially can reduce access to prenatal sex determination, although in practice its impact is thought to remain limited in North India (eg, UNFPA 2014).
