# Supplementary material for: Is sex ratio at birth an appropriate measure of prenatal sex selection? Findings of a theoretical model and its application to India
Source: BMJ Glob Health. 2018 Jul 19;3(4):e000675. doi: 10.1136/bmjgh-2017-000675 (PMC6058172; doi:10.1136/bmjgh-2017-000675)
Supplement: Supplementary data [file bmjgh-2017-000675supp003.pdf]

Appendix: *a simple son-preference model*

Our aim was to construct a model which captured the essence of a son-preference tendency while being simple enough to be amenable to analytical investigation. To this end, we began by considering the following hypothetical scenario: a particular community, characterized by an average number of children per family, λ, all exercise an innate desire for a male offspring according to the rule that families which reach (or exceed) some critical number of children, n, invoke medical intervention if necessary to ensure that at least one child is a boy. This is formally specified through the following (conditional) probability assignments.

The probability that a couple has N children, given that the average across families is λ, is taken to be a Poisson distribution:

$$\mathbb{P}(N|\lambda) = \frac{\lambda^N e^{-\lambda}}{N!} \tag{1}$$

for $N = 0, 1, 2, 3, \ldots$. In the absence of any gender bias (or identical siblings), and with the natural chance of a female birth being p, the probability of a couple with N children having G girls is given by the binomial distribution:

$$\mathbb{P}(G|N, p, \text{no bias}) = \frac{N!}{G!\,(N-G)!}\; p^G\,(1-p)^{N-G} \tag{2}$$

for $G = 0, 1, 2, \ldots, N$ and $0 \leqslant p \leqslant 1$. The well known result about the mean of a binomial distribution leads to the useful formula

$$\mathbb{E}(G|N, p, \text{no bias}) = \sum_{G=0}^{N} G\;\mathbb{P}(G|N, p, \text{no bias}) = Np \tag{3}$$

for the expected number of girls, with the corresponding figure for boys being $N(1-p)$. The final ingredient of our universal son-preference model, and the one which defines its essential character, is the proposition that medical intervention is invoked if necessary to ensure at least one male offspring if the total number of children in a household reaches a certain threshold, n. This means that

$$\mathbb{P}(G|N<n, p) = \mathbb{P}(G|N, p, \text{no bias}) \tag{4}$$

but a procedure is used to ensure that the nth child is a boy if $N \geqslant n$ and the first $n-1$ births have all been girls; no further bias is assumed for $N > n$.

The expected number of girls in a family under this model, when given λ, p and n, can be calculated by using standard results from probability theory. From the partition theorem, for example,

$$\mathbb{E}(G|\lambda, p, n) = \sum_{N=0}^{\infty} \mathbb{E}(G|N, p, n)\;\mathbb{P}(N|\lambda) \tag{5}$$

where the conditioning on λ has been dropped from the first term in the summation, and p and n have been omitted (as given) in the second, for simplicity, due to their irrelevance for G and N respectively. While $\mathbb{P}(N|\lambda)$ is defined for all natural values of N by eqn (1), the expected number of girls is only specified for $N < n$ by eqns (3) and (4):

$$\mathbb{E}(G|N<n, p) = Np\;\cdot \tag{6}$$

To evaluate eqn (5), therefore, we must ascertain $\mathbb{E}(G|N \geqslant n, p)$. The easiest way of doing so is to consider the couples with N children that would naturally have had all girls for the first n of them, for they will now have one less girl than they would have otherwise (the nth child having to be a boy). Since they constitute a fraction p^n of the relevant families,

$$\mathbb{E}(G|N \geqslant n, p) = Np - p^n\;\cdot \tag{7}$$

Hence, eqn (5) becomes

$$\mathbb{E}(G|\lambda,p,n) \,=\, p\sum_{N=0}^{\infty} N\ \mathbb{P}(N|\lambda) \,-\, p^n \sum_{N=n}^{\infty} \mathbb{P}(N|\lambda)\,\cdot \tag{8}$$

The first summation on the right-hand side is simply the mean value of N for the Poisson distribution of eqn (1), namely λ, while the second is constrained by probability normalization. Thus,

$$\mathbb{E}(G|\lambda,p,n) \,=\, \lambda\, p - p^n \left[1 - \mathrm{e}^{-\lambda} \sum_{N=0}^{n-1} \frac{\lambda^N}{N!}\right]\cdot \tag{9}$$

Finally, the sex ratio at birth, R, of the expected number of boys (B) to the expected number of girls can be calculated through

$$R \,=\, \frac{\mathbb{E}(B|\lambda,p,n)}{\mathbb{E}(G|\lambda,p,n)} \,=\, \frac{\lambda - \mathbb{E}(G|\lambda,p,n)}{\mathbb{E}(G|\lambda,p,n)} \tag{10}$$

where the fact that the average number of children per family, boys plus girls, is λ has been used in the numerator.

The proportion of couples invoking gender selective intervention, ϕ, is given by

$$\phi \,=\, p^{n-1} \left[1 - \mathrm{e}^{-\lambda} \sum_{N=0}^{n-1} \frac{\lambda^N}{N!}\right] \tag{11}$$

and follows from the probability that a couple will have n or more children and that the first $n-1$ of them will all be girls. Strictly speaking, this assumes an IVF-type procedure to ensure that the nth child of such couples is a boy. If it is based on ultrasound sex-determination and subsequent abortion, it could be argued that only about half the fraction given in eqn (11) actually engage in gendercide. In that case, half of them will have to abort for a second time, and half of those for a third time, and so on, to ensure that their n^{th} child is male. Since the sum of the geometric series

$$p + p^2 + p^3 + p^4 + \,\cdots\, \,=\, \frac{p}{1-p} \,\approx 1 \ \text{ for } p \approx 0{\cdot}5\,, \tag{12}$$

eqn (11) is a good measure of the number of sex-selective procedures in either case.

As the fertility rate decreases, the proportion of childless couples increases. Taking them out of the intervention equation, the proportion of parents invoking a gender selective procedure, ψ, is related to eqn (11) through

$$\psi \,=\, \frac{\phi}{1 - \mathrm{e}^{-\lambda}} \tag{13}$$

with $\lambda > 0$.

Incorporating a male-oriented stopping rule

Demographic studies have shown that a higher than expected proportion of couples with three children have two initial offspring that are of the same gender. The sex seems immaterial in Western cultures, but is predominantly female in the case of Asian families. Such observations are indicative of a desire for a male offspring in Eastern cultures, whereas Western societies display a preference for having a child of each gender.

Although Indian couples with two girls may be more likely to try and conceive a third child than those with a male or mixed gender pair, in the hope of having at least one boy, this will not of itself lead to any imbalance from the

natural ratio of around 106 boys for every 100 girls at birth. The latter requires some actual intervention to ensure that the additional offspring is of the desired gender. We now consider a modification of the above model to mimic the male-oriented stopping rule.

The difference between the earlier analysis, and the one presented below, hinges on the significance of the intervention threshold, n. Previously, this represented the child order at which a male-selective procedure was used if all the offspring had thus far been female. Such an intervention is now invoked for the **last** child in a family, when all the other offspring are female, but only if the order of the final birth, N, equals or exceeds n. Technically, following the binomial assignment of eqn (2), this means that

$$\mathbb{P}(\text{intervention}|N,p,n) \;=\; \begin{cases} p^{N-1} & \text{for } N \geqslant n\,, \\ 0 & \text{otherwise}\,. \end{cases} \tag{14}$$

Using the marginalisation and product rules of probability, the fraction of couples that will invoke a sex-selective intervention is then given by

$$\phi \;=\; \mathbb{P}(\text{intervention}|\lambda,p,n) \;=\; \sum_{N=n}^{\infty} p^{N-1}\,\mathbb{P}(N|\lambda) \tag{15}$$

where λ is the average number of children per family. For the Poisson distribution of eqn (1), this sum yields

$$\phi \;=\; \frac{\mathrm{e}^{-\lambda}}{p}\left[\mathrm{e}^{\lambda p} - \sum_{N=0}^{n-1}\frac{(\lambda p)^N}{N!}\right]. \tag{16}$$

As explained below, this intervening fraction can be related directly to the ratio of the expected number of boys to that of girls at birth through the fertility rate:

$$R = \frac{R_0\,\lambda + \phi}{\lambda - \phi} \tag{17}$$

where $R_0 = (1-p)/p \approx 1{\cdot}06$ for $p = 0{\cdot}486$.

On average, each couple has λ children. Of these, $\lambda(1-p)$ are expected to be boys and λp girls. If the probability of a gender selective intervention is ϕ, this will result in ϕp fewer girls and, correspondingly, ϕp more boys. The boy-to-girl sex ratio at birth will then be

$$R = \frac{\lambda(1-p) + \phi p}{\lambda p - \phi p}\,. \tag{18}$$

Equation (17) follows readily. It can easily be rearranged, of course, to express the intervening fraction in terms of the ratio of the expected number of boys to that of girls at birth and the fertility rate:

$$\phi \;=\; \frac{(R-R_0)\,\lambda}{1+R}\,. \tag{19}$$
