# Supplementary figures and images for: Is sex ratio at birth an appropriate measure of prenatal sex selection? Findings of a theoretical model and its application to India
Source: BMJ Glob Health. 2018 Jul 19;3(4):e000675. doi: 10.1136/bmjgh-2017-000675 (PMC6058172; doi:10.1136/bmjgh-2017-000675)

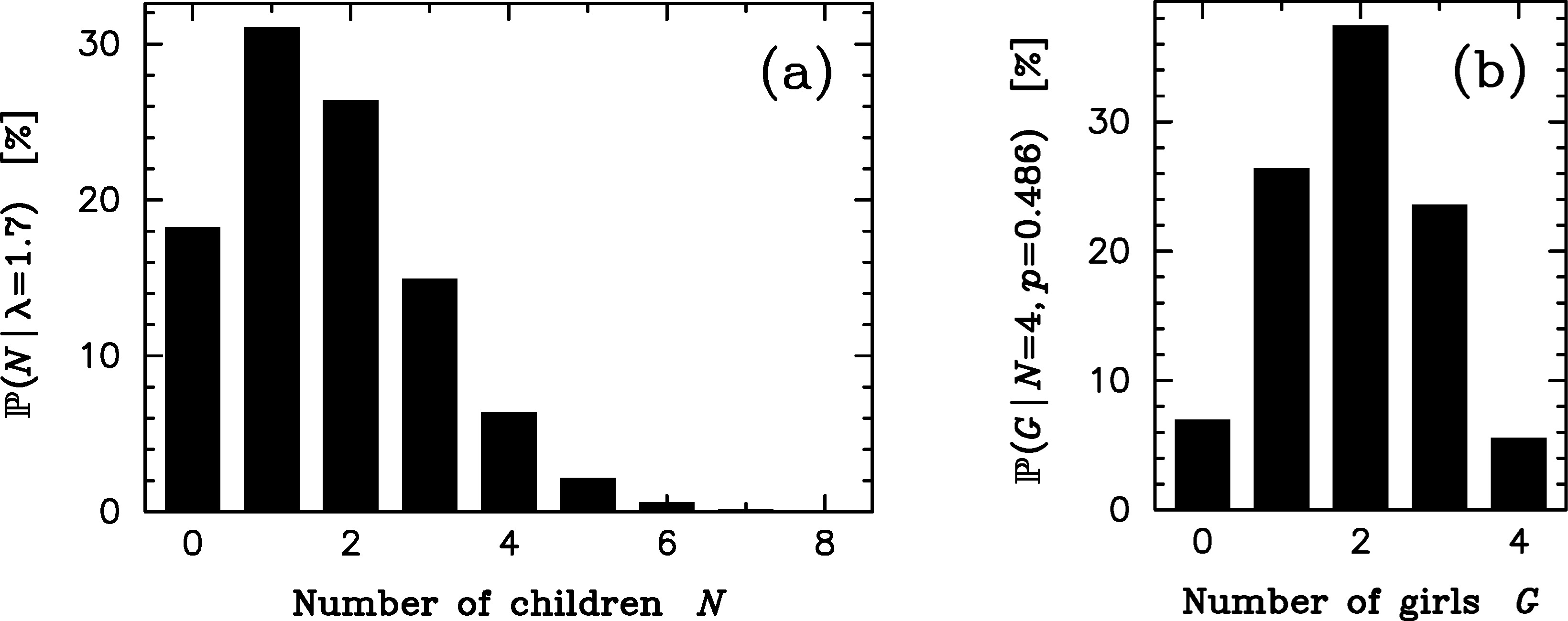

Supplement: Supplementary data [file bmjgh-2017-000675supp001.jpg]

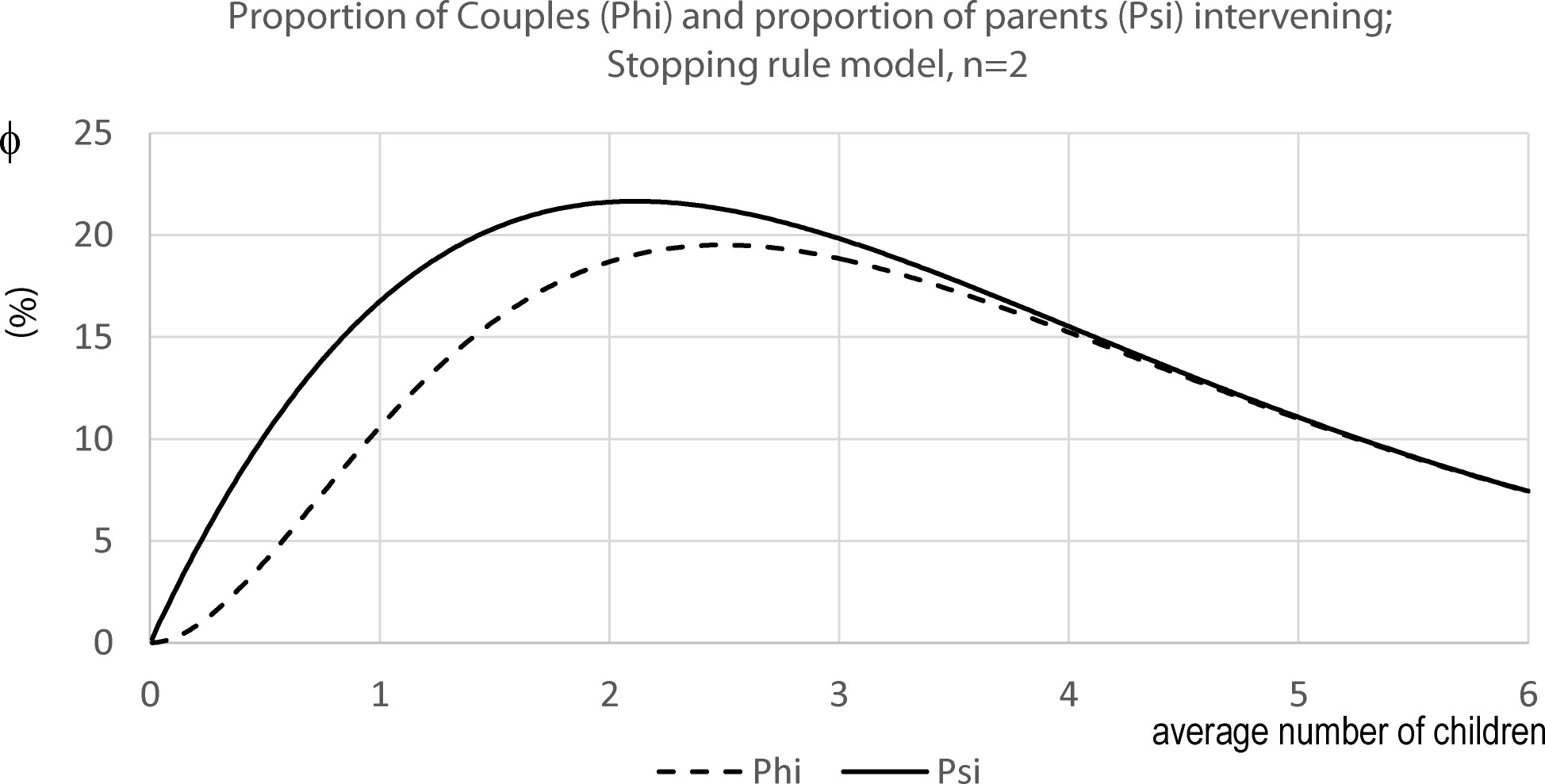

Supplement: Supplementary data [file bmjgh-2017-000675supp002.jpg]
